# Appropriate Body Mass Index and Waist-hip Ratio Cutoff Points for Overweight and Obesity in Adults of Northeast China

**Published:** 2017-08

**Authors:** Qiong YU, Bo PANG, Rui LIU, Wenwang RAO, Shangchao ZHANG, Yaqin YU

**Affiliations:** Dept. of Epidemiology and Biostatistics, School of Public Health, Jilin University, Changchun, Jilin, China

**Keywords:** Body mass index, Waist-hip ratio, Adult, Cutoff point, China

## Abstract

**Background:**

The current overweight and obesity guidelines based on the Westerners are not consistent with many studies based on the Asians. The guidelines may be different because of regional diversity. This study aimed to determine the appropriate body mass index (BMI) and waist-hip ratio (WHR) cutoff points in the adults of Northeast China.

**Methods:**

Overall, 21206 adults were selected from Jilin Province Adult Chronic Disease and Risk Factor Survey conducted in 2012. A representative sample was collected in the Jilin Province of northeast China by a multistage stratified random cluster sampling design. The age of participants was from 20 to 79 yr old. The test items were clustered by risk factors, and receiver operating characteristic (ROC) curves were computed to analyze.

**Results:**

Under different risk factors, BMI cutoff points were affected greatly. Especially for diabetes, the cutoff value was apparently larger than others were. WHR increased with age in both genders. From a general view, male WHR was slightly larger than female. In the male, WHR cutoff point was near 0.88 with a tiny change, as for in the female was near 0.86.

**Conclusion:**

The cutoff values of sensitivity and specificity are relatively good and false positives rate is relatively low. BMI cutoffs values of overweight and obesity are 24.5 kg/m^2^ and 29.0 kg/m^2^, WHR cutoff values of the male are 0.88, the female is 0.86.

## Introduction

Overweight and obesity, which broadly refer to access the acceptable or desirable weight, have become important public health problems. Overweight and obesity had close with diseases which involve hypertension (1), diabetes (2), hyperlipidemia (3) and so on (4, 5). Hence, preventing overweight and obesity has been the key point to control chronic diseases.

In order to predict risks of cardiovascular disease, Body Mass Index (BMI) and Waist-Hip Ratio (WHR) have been widely used to judge overweight and obesity (6–8). In epidemiology, BMI is used as the measurement of overall obesity, and WHR is the measurement of central obesity. Using the data of Westerners, WHO has classified BMI between 18.5–24.9 kg/m^2^ as normal weight, between 25.0–29.9 kg/m^2^ as overweight, and ≥30.0 kg/m^2^ as obese (9). In 2002, based on the data of each province of China, China Working Group on Obesity (WGOC) suggested classifying BMI between 24.0–28.0 kg/m^2^ as overweight, and ≥ 28.0 kg/m^2^ as obese in Chinese (10). However, because of the wide geographic span in China, there are significant differences in lifestyle, dietary habit, and hereditary factors etc. Compared with southerners, the northeasterners located in the North Temperate Zone, show obvious gaps in physical figure and function. Therefore, in authors’ opinion, BMI cutoff values that WGOC classified may not accurately describe the condition of northeasterners in China. At present, the specific measurement of WHR has not been given in China.

It is imperative to determine a more appropriate BMI or WHR range and the threshold of overweight and obesity for more effective prevention and control of complications of obesity in northeastern adults. In addition to determining the cutoff points, the study will analyze their associations with related disease risk factors for adults aged 20 to 79 yr in northeast China.

## Methods

### Ethics statement

This study was approved by the Ethics Committee of School of Public Health Jilin University and in accordance with the Helsinki Declaration. Anonymity and privacy of respondents were fully guaranteed. Participants had right to quit from the research at any time without any reason or explanation. The informed consent was obtained from each participant.

### Study population

There are three provinces from north to south in northeast China. Jilin Province is located in the middle of northeast China and has a population of about 27 million people. Therefore, in consideration of representativeness, this study used data from Jilin Province Investigation Team on Adult Chronic Disease and Risk Factor. Jilin Province Adult Chronic Disease and Risk Factor Survey was conducted by the Department of health, Jilin Province and was led by a research team from the School of Public Health, Jilin University in Jun 2012. Totally, 22600 populations, which was usually adult residents from 32 survey spots covering 9 cities of Jilin Province, was investigated with multistage stratified random cluster sampling method. Of those, 21206 individuals aged 20 to 79 were selected in this study. The survey respondents were asked to answer each question face-to-face with investigators uniformly trained.

### Anthropometric measurements and laboratory methods

#### BMI and WHR:

With a precision of 0.1kg and 0.1cm, weight, height, waist circumference (WC) and hip circumference (HC) were measured 2 times in light indoor clothing and without shoes. At the level of the abdomen just above the hip-bone, the WC measurement was taken immediately after exhalation. The HC measurement was at the level of the symphysis pubis and the place most protruding of the hip. The average of two measurements was used for analysis. BMI is calculated as weight in kilogram divided by height in m^2^ and WHR is the WC divided by the HC.

#### Blood pressure:

At the sitting position, blood pressure was measured 2 times on the left arm. The first measurement was made after at least 5 min rest and the second measurement was taken after 2 min’ interval (11).

#### Blood lipids:

Blood samples were drawn from the antecubital vein into vacuum tubes containing EDTA in the morning after an overnight fasting period. All the collected samples were transported on dry ice at prearranged intervals to KingMedDiagnostics, a commercial clinical laboratory in China. The blood lipid was measured by Roche MODULE P800 Automatic Biochemical Analyzer (Roche Holding AG, America).

#### Blood glucose:

Using a drop of blood from participants’ finger, blood glucose was measured by Bayer Contour TS Blood Glucose Meter (Bayer, Germany).

### Definition of risk factors

#### Hypertension:

Hypertension was defined as systolic blood pressure (SBP), calculated from the mean of the two readings, ≥140 mmHg or diastolic blood pressure (DBP) ≥90 mmHg (11). Participants who were currently on anti-hypertensive medication or diagnosed by the hospital above the county level were also classified as hypertensive cases.

#### Hyperlipidemia:

Hyperlipidaemia was defined as total cholesterol (TC) concentration ≥ 5.18 mmol/L, triglyceride (TG) concentration ≥ 1.70 mmol/L, LDL cholesterol (LDL-C) concentration ≥3.37 mmol/L or HDL cholesterol (HDLC) concentration ≤1.04 mmol/L (12). Participants who were currently on anti-hyperlipidemia medication or diagnosed by the hospital above the county level were also classified as hyperlipidemia cases.

#### Diabetes:

According to the criteria for the diagnosis of diabetes mellitus reported by the expert committee of ADA and a WHO working group, diabetes was defined as fasting plasma glucose ≥7.0 mmol/L, postprandial blood glucose ≥11.1 mmol/L, using of insulin or oral hypoglycemic agents, or a self-reported history of diabetes. Participants diagnosed by the hospital above the county level were also classified as diabetes cases (13, 14).

#### Clustering of risk factors:

Two or more risk factors were included.

### Statistical analysis

All records were entered into EpiData version 3.1 and analyzed using SPSS version 18.0 (Chicago, IL, USA) for Windows at two-sided 5% significance level. Continuous variables were expressed as means and standard deviations, and discrete variables were expressed as quantities and proportions. Data were classified by gender and age standardized using the 2010 Jilin provincial population as the standard population. It was stratified to 12 subgroups at 20-, 30-, 40-, 50-, 60-, 70–79 yr. The Receiver Operating Characteristic (ROC) curves of BMI and WHR were drawn and the areas under the curve (AUC), 95% Confidence Interval (95%CI), sensitivity (Sens), specificity (Spec), positive likelihood ratio (PLR) and negative likelihood ratio (NLR) were tabulated to judge cutoff points. The cutoff points of WHR and overweight points of BMI were judged according to Max Youden Index (YI, equal to Sens+Spec−1) and obesity points of BMI were the point that Spec ≥ 90% (10, 15). Additionally, the cutoff value of subgroups of BMI and WHR were also calculated to observe trends.

## Results

Anthropometric measurement data and risk factors prevalence of the study subjects is shown in Table 1. Overall, 21206 adults participated in the study, of whom 48.07% (10193) were female, with a mean age of 48.14± (13.65) yr. In the study, females are older than males (48.14± (13.65) vs 46.55± (12.61) yr old; *P*<0.001).

**Table 1:** Anthropometric measurement data and risk factors prevalence

	**Age (yr)**	**Sample (n)**	**BMI (mean ± sd)**	**WHR (mean ± sd)**	**Hypertension n(%)**	**Hyperlipidaemia n(%)**	**Diabetes n(%)**	**Clustering of risk factors**
Male	20∼	1371	23.6±4.4	0.84±0.06	165(12.0)	444(32.4)	13(0.9)	92(6.7)
	30∼	1822	24.7±4.0	0.88±0.06	406(22.3)	915(50.2)	66(3.6)	311(17.1)
	40∼	2697	24.8±3.6	0.89±0.06	1039(38.5)	1514(56.1)	246(9.1)	830(30.8)
	50∼	2386	24.4±3.3	0.90±0.06	1167(48.9)	1389(58.2)	332(13.9)	911(38.2)
	60∼	1421	24.0±3.4	0.90±0.06	795(55.9)	809(56.9)	232(16.3)	600(42.2)
	70∼79	496	23.6±3.5	0.91±0.07	311(62.7)	276(55.6)	83(16.7)	218(44.0)
	Total	10193	24.4±3.7	0.89±0.07	3883(38.1)	5347(52.5)	972(9.5)	2962(29.1)
Female	20∼	915	21.8±3.6	0.79±0.06	34(3.7)	160(17.5)	8(0.9)	19(2.1)
	30∼	1792	23.2±3.7	0.81±0.06	158(8.8)	504(28.1)	39(2.2)	92(5.1)
	40∼	3395	24.3±3.6	0.84±0.07	874(25.7)	1509(44.4)	172(5.1)	585(17.2)
	50∼	2713	24.8±3.6	0.87±0.06	1190(43.9)	1827(67.3)	350(12.9)	1013(37.3)
	60∼	1696	24.9±3.7	0.89±0.07	1014(59.8)	1196(70.5)	321(18.9)	864(50.9)
	70∼79	502	25.1±2.8	0.92±0.06	344(68.5)	366(72.9)	94(18.7)	290(57.8)
	Total	11013	24.1±3.7	0.85±0.07	3614(32.8)	5562(50.5)	984(8.9)	2863(26.0)

For both female and male, the prevalence of hypertension obviously raised with the increase of age. Under sixty, male patients were more than female patients were, and over sixty is opposite. Likewise, the prevalence of hyperlipidemias and diabetes is higher with higher age, which rises quickly and then levels off.

### Body Mass Index

In male adults, mean of BMI began to rise and it was a peak in the group 40-year-old, and then declines from the peaks. In the female, BMI rises continuously with age, meanwhile, after 40 yr old, BMI is larger than male (Table 1).

**Fig. 1:**
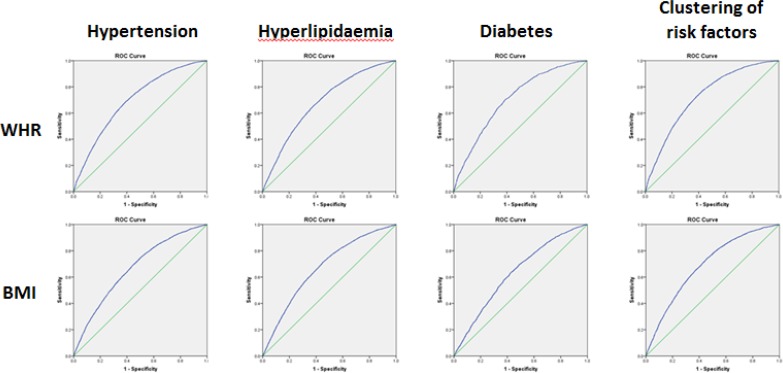
ROC curves for BMI and WHR across all the samples

**Fig. 2:**
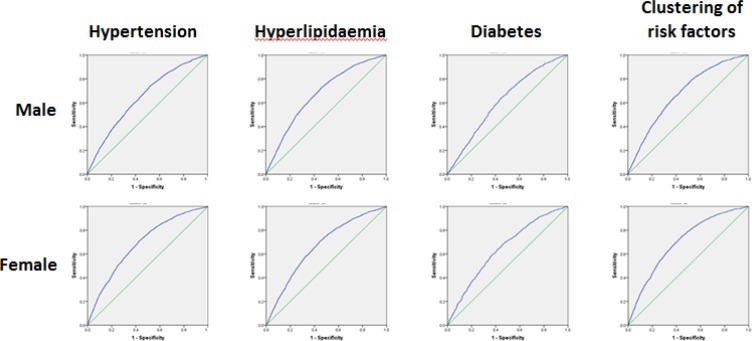
ROC curves for BMI across the subgroups of samples

The result of ROC curves for BMI cutoffs across all the samples was presented in Table 2 and Fig. 1. Cutoff points whose YI are largest are chosen. Under the different risk factors, BMI was affected greatly. Especially for diabetes, the cutoff point was apparently larger than others were. Categorized by age and gender, a series of ROC curves were obtained (Table 3 and Fig. 2).

**Table 2:** The result of ROC curves for BMI and WHR cutoffs across all the samples

		**AUC(95%CI)**	***P* value**	**Sens**	**Spec**	**PLR**	**NLR**	**Cutoff**
WHR	Hypertension	0.668 (0.657–0.678)	<0.001	0.688	0.568	1.59	0.55	0.88
	Hyperlipidaemia	0.674 (0.664–0.685)	<0.001	0.686	0.575	1.61	0.55	0.88
	Diabetes	0.679 (0.662–0.697)	<0.001	0.722	0.567	1.67	0.49	0.89
	Clustering of riskfactors	0.726 (0.718–0.733)	<0.001	0.767	0.576	1.81	0.41	0.86
BMI	Hypertension	0.647 (0.636–0.658)	<0.001	0.735	0.484	1.42	0.55	23.2
	Hyperlipidaemia	0.674 (0.663–0.684)	<0.001	0.723	0.541	1.58	0.51	23.2
	Diabetes	0.618 (0.601–0.636)	<0.001	0.629	0.563	1.44	0.66	24.6
	Clustering of risk factors	0.689 (0.682–0.697)	<0.001	0.741	0.543	1.62	0.48	23.7

**Sens**: sensitivity; **Spec**: specificity; **PLR**: positive likelihood ratio; **NLR**: negative likelihood ratio.

**Table 3:** The result of ROC curves for BMI cutoffs across the subgroups of samples

	**Age**	**Male**				**Female**			
		**AUC(95%CI)**	***P*-value**	**Overweight**	**Obesity**	**AUC(95%CI)**	***P*-value**	**Overweight**	**Obesity**
Hypertension	20∼	0.691 (0.668–0.715)	<0.001	24.1	28.8	0.724 (0.685–0.763)	<0.001	24.5	27.3
	40∼	0.655 (0.640–0.671)	<0.001	23.8	27.9	0.673 (0.659–0.688)	<0.001	23.8	28.0
	60∼79	0.643 (0.618–0.669)	<0.001	22.1	27.1	0.620 (0.595–0.644)	<0.001	23.6	28.3
	**Total**	0.647 (0.636–0.658)	<0.001	**23.2**	**28.2**	0.686 (0.676–0.697)	<0.001	**23.7**	**27.8**
Hyperlipidaemia	20∼	0.699 (0.680–0.718)	<0.001	23.1	28.2	0.682 (0.658–0.705)	<0.001	22.8	26.8
	40∼	0.665 (0.649–0.680)	<0.001	23.8	27.9	0.640 (0.626–0.654)	<0.001	24.3	28.0
	60∼79	0.648 (0.623–0.673)	<0.001	22.8	27.2	0.597 (0.570–0.624)	<0.001	22.6	28.7
	**Total**	0.674 (0.663–0.684)	<0.001	**23.2**	**27.9**	0.667 (0.656–0.667)	<0.001	**23.2**	**27.7**
Diabetes	20∼	0.652 (0.598–0.706)	<0.001	22.7	29.5	0.679 (0.596–0.761)	<0.001	25.0	27.6
	40∼	0.627 (0.603–0.651)	<0.001	24.6	28.8	0.627 (0.602–0.652)	<0.001	25.1	28.9
	60∼79	0.609 (0.576–0.642)	<0.001	23.7	28.2	0.585 (0.556–0.615)	<0.001	24.9	29.1
	**Total**	0.618 (0.601–0.636)	<0.001	**24.6**	**28.9**	0.645 (0.627–0.662)	<0.001	**24.5**	**28.7**
Clustering of Risk factors	20∼	0.719 (0.694–0.744)	<0.001	24.5	29.1	0.756 (0.708–0.805)	<0.001	24.6	27.4
	40∼	0.682 (0.667–0.698)	<0.001	25.0	28.0	0.693 (0.679–0.708)	<0.001	25.0	28.2
	60∼79	0.679 (0.655–0.703)	<0.001	23.2	27.2	0.633 (0.609–0.656)	<0.001	23.6	28.5
	**Total**	0.677 (0.666–0.688)	<0.001	**23.2**	**28.3**	0.700 (0.689–0.711)	<0.001	**23.7**	**28.0**

Points of largest YI were chosen as the overweight points and the points of Spec ≥ 90% were chosen as the obesity points. In consideration of reliability of data and being easy to memorize, authors suggest determining 24.5 as the overweight cutoff points and 29.0 as the obesity points.

### Waist-to-hip ratio

**Fig. 3:**
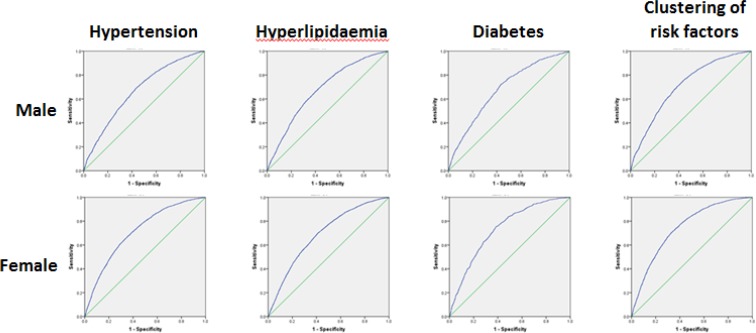
ROC curves for WHR across the subgroups of samples

WHR increased with age in both genders. From a general view, male WHR was slightly larger than female (Table 1). The WHR cutoff points with largest YI were chosen (Table 2 and 4, Fig. 1 and 3). In the male, WHR was near 0.88 with tiny changing, as for female near 0.86. Therefore, authors suggested 0.88 and 0.86 as the WHR cutoff values of male and female respectively (Table 5).

**Table 4:** The result of ROC curves for WHR cutoffs across the subgroups of samples

	**Age (yr)**	**Male**			**Female**		
		**AUC(95%CI)**	***P***-value	**Cutoff point**	**AUC(95%CI)**	***P*-value**	**Cutoff point**
Hypertension	20∼	0.667 (0.642–0.691)	<0.001	0.88	0.708 (0.670–0.747)	<0.001	0.82
	40∼	0.639 (0.623–0.655)	<0.001	0.88	0.656 (0.641–0.670)	<0.001	0.87
	60∼79	0.668 (0.657–0.678)	<0.001	0.88	0.714 (0.704–0.724)	<0.001	0.85
	**Total**	0.706 (0.649–0.763)	<0.001	**0.88**	0.686 (0.676–0.697)	<0.001	**0.87**
Hyperlipidaemia	20∼	0.694 (0.675–0.714)	<0.001	0.85	0.656 (0.633–0.680)	<0.001	0.80
	40∼	0.664 (0.648–0.680)	<0.001	0.87	0.646 (0.632–0.660)	<0.001	0.84
	60∼79	0.634 (0.608–0.660)	<0.001	0.91	0.590 (0.563–0.617)	<0.001	0.87
	**Total**	0.674 (0.664–0.685)	<0.001	**0.88**	0.690 (0.680–0.699)	<0.001	**0.84**
Diabetes	20∼	0.652 (0.598–0.708)	<0.001	0.88	0.731 (0.655–0.809)	<0.001	0.84
	40∼	0.660 (0.636–0.683)	<0.001	0.89	0.708 (0.685–0.730)	<0.001	0.86
	60∼79	0.621 (0.587–0.654)	<0.001	0.89	0.604 (0.575–0.633)	<0.001	0.88
	**Total**	0.679 (0.662–0.697)	<0.001	**0.89**	0.733 (0.717–0.748)	<0.001	**0.86**
Clustering of Risk factors	20∼	0.712 (0.685–0.738)	<0.001	0.88	0.767 (0.727–0.807)	<0.001	0.83
	40∼	0.686 (0.671–0.702)	<0.001	0.89	0.702 (0.688–0.716)	<0.001	0.86
	60∼79	0.675 (0.650–0.699)	<0.001	0.90	0.618 (0.594–0.641)	<0.001	0.88
	**Total**	0.708 (0.697–0.719)	<0.001	**0.88**	0.744 (0.734–0.754)	<0.001	**0.86**

**Table 5:** The suggestion of appropriate BMI and WHR cutoffs in Northeast China

**BMI**		**WHR**	
Overweight	Obesity	Male	Female
24.5kg/m^2^	29.0kg/m^2^	0.88	0.86

## Discussion

To our knowledge, this was the first study to examine the appropriate BMI and WHR cutoff for overweight and obesity among adults in northeast China. However, in other areas, there have been many studies about the predictive value of BMI and WHR to multiple risk factors (16–21). There are different optimal BMI or WHR cutoffs in different populations. The relationship of BMI or WHR with risk factors is approximately continuous linearity without significantly fold point or threshold, so it is hard to avoid quite a part of overlap between population at high risk and the healthy. Thus, all the cutoffs are artificial and relative but scientific.

The objective of the study is to provide a theoretical basis for designing preventive measures and treatments of overweight and obesity. For above purpose, the principle of choosing cutoff values is that the cutoffs not only can serve, as a reliable index to prevent the increase of most chronic disease early, but also will not give public too much mental pressure, and reduce the burden of preventions and treatments. Therefore, authors chose the points of which Sens and Spec are relatively well and false positive rate is relatively low. Comparing the analysis results with the standard WGOC classified in 2002 (BMI between 24.0–28.0 kg/m^2^ as overweight, and ≥28.0 kg/m^2^ as obesity), both the overweight and obesity cutoffs are higher than the average level in China. In theory, the northeastern population is stronger and has a lower risk for chronic diseases such as hypertension than the southern population with the same BMI. However, prevalence rate of hypertension (35.4% VS 26.6 % (22)) is significantly higher than the average level in China and the situation of diabetes (9.68% VS 11.6% (23)) is optimistic. The authors consider the phenomenon may be related to the cold environment, dietary habit, aware of hypertension prevention and hereditary factors. Among them, inheritance may play a key role, reported in domestic and overseas literature (24–27).

Analyzing the data of ROC curves, the cutoffs of Hypertension and clustering of risk factors continuously decline with age. Therefore, the elderly should attach great importance to keep normal weight. As for diabetes and hyperlipidemia, they should be paid attention to as a youth. Although there are some differences on the numerical between male and female subgroups, trends with the age are identical.

Because of the relatively low Sens and Spec in this study, the results have a few limitations. Cutoffs should be used as screening criteria among northeastern Chinese, and diseases diagnosis would still need a further examination.

## Conclusion

The suggestion of appropriate BMI cutoffs measuring overall obesity and waist-hip ratio cutoffs measuring central obesity in Northeast China are listed in Table 5. Our findings would be useful for policy makers for the development of strategies to prevent chronic diseases that are gradually increasing in China.

## Ethical considerations

Ethical issues (Including plagiarism, informed consent, misconduct, data fabrication and/or falsification, double publication and/or submission, redundancy, etc.) have been completely observed by the authors.
